# Leptin in depression: a potential therapeutic target

**DOI:** 10.1038/s41419-018-1129-1

**Published:** 2018-10-26

**Authors:** Tongtong Ge, Jie Fan, Wei Yang, Ranji Cui, Bingjin Li

**Affiliations:** Jilin Provincial Key Laboratory on Molecular and Chemical Genetics, The Second Hospital of Jilin University, 130041 Changchun, Jilin China

## Abstract

Leptin, produced and secreted by white adipose tissue, plays a critical role in regulating body weight, food intake, and energy metabolism. Recently, several studies have identified an underlying role for leptin in regulation of mood and cognition via regulation of synaptic changes in the brain that have been associated with antidepressant-like actions. Brain neural plasticity occurs in response to a range of intrinsic and extrinsic stimuli, including those that may mediate the effects of antidepressants. Neural plasticity theories of depression are thought to explain multiple aspects of depression and the effects of antidepressants. It is also well documented that leptin has effects on neural plasticity. This review summarizes the recent literature on the role of leptin in neural plasticity in order to elaborate the possible mechanism of leptin’s antidepressant-like effects. Recent findings provide new insights into the underlying mechanisms of neural plasticity in depression. Leptin may influence these mechanisms and consequently constitute a possible target for novel therapeutic approaches to the treatment of depression.

## Facts

- Leptin, produced and secreted by white adipose tissue, is a member of the class of hormones called adipokines.
- Leptin can affect mood and cognition via inducing structural and functional alterations in hippocampus and prefrontal cortex.
- Neural plasticity theories of depression may explain multiple aspects of brain plasticity changes associated with depression and antidepressant-like effects.

## Open questions

- Can the decrement of peripheral leptin during the development of depression constitute a biomarker for clinical diagnosis?
- How does leptin act as a neurotrophic factor and exert neuroprotective actions in depression?
- What role does metabolism play in the physiological and pathological changes of depression and other mental illnesses?

## Introduction

Depression is a highly prevalent and devastating mood disorder worldwide that exacts a serious physical and psychological burden on patients and their families. Numerous genetic, endocrine, environmental factors, and the interactions between those factors, may affect the progression of depressive disorders and contribute to individual susceptibility^1,2^. However, the underlying etiology and pathophysiology of depression remains incompletely understood. Traditional theories regarding the pathological mechanisms of depression implicate the disruption of neurotrophic factor regulation, monoamine neurotransmission and hippocampal neurogenesis^3,4^. Clinical data indicate that currently available treatments for depression possess various limitations in therapeutic efficacy and onset time^5,6^. Typical antidepressants treatments take several weeks or even months to achieve remission of depressive symptoms. Serious side effects, including acute nausea, sexual dysfunction, and weight gain, limit treatment efficacy^7,8^. Thus, identification of novel therapeutic targets based upon newer understanding of the etiology of affective disorders would greatly improve treatment.

Leptin is a 167 amino acid polypeptide product of OB gene produced and secreted by white adipose tissue^9^. Leptin can cross the blood brain barrier (BBB) and binds to a specific leptin receptor (LepRb)^10^ that is distributed in several specific brain regions, including several hypothalamic and thalamic regions, the substantia nigra pars compacta and the cerebellum, with lower levels of expression in some cortical areas and other brain regions. Leptin is an established and critical regulator of feeding behavior and energy expenditure^11^, but the brain distribution of LepRb also suggests that it may be involved in other neural functions. Several basic studies suggest that leptin has antidepressant effects and might be a potential therapeutic target for depression. LepRb deficiency leads to memory and cognitive impairments that are accompanied by alterations in hippocampal synaptic plasticity^12^. Acute administration of leptin has been reported to have antidepressant and anxiolytic effects in mice^13^. In addition, clinical studies have shown that the expression of leptin mRNA and protein are positively associated with depression severity. In this review, we present the available evidence, and try to illuminate the potential cellular and molecular mechanisms of leptin’s antidepressant actions and its potential therapeutic capacity in the treatment of depression.

## Mechanisms of neural plasticity in depression

The term “neural plasticity” represents the ability of a neuron or the brain to adapt to external or internal stimuli, involving a series of cellular events, including neurogenesis, cell migration, cell survival, synaptogenesis, and the modification of mature synapses^14–16^. Although such changes are generally adaptive, they may also be maladaptive under some conditions. Adaptive neuroplasticity in response to stimuli that evoke depressive symptomatology, including chronic stress, include both functional and structural changes, detected as neuroanatomical, neurochemical, and electrophysiological alterations^17^. Long-term exposure to stress and depression can result in neuronal atrophy, dendrite remodeling and synaptic dysfunctions in hippocampus, amygdala, and the prefrontal cortex (PFC) as shown in both in human and animal studies. Typical antidepressant drugs partly lead to reversal of stress-induced alterations in neuroplasticity^15^. This evidence provides support for the neuroplasticity hypothesis of depression. Regulation of neural plasticity appears to be involved in the therapeutic mechanisms of available antidepressant therapies, but more work remains to be done to demonstrate the necessity and importance of these changes.

### Dysfunction of neuronal morphology in depression

Long-lasting alterations in cellular morphology, affecting cellular function and plasticity, may contribute to depression and other mood disorders^18^. Patients with major depressive disorder (MDD) have reduced neuronal density in the PFC, reduced synapse numbers, and reduced dendritic complexity compared to unaffected controls^19^. These changes in cellular morphology are consistent with the finding of structural atrophy in the hippocampus and PFC of depressed patients^20,21^. Clinical magnetic resonance imaging (MRI) studies show that patients with depression have reduced hippocampal volumes^22–24^, although this is not universally found^25^. Gerritsen et al. suggested that the amygdala:hippocampus volume ratio, rather than hippocampus or amygdala volume along is a better indicator of depression risk^26^. Moreover, there are positive associations between clinical improvement of depressive symptoms and hippocampal volume^27^. Additionally, depressive behavioral deficits induced by chronic stress correlate with the reduced number, and loss of function, of spine synapses in the PFC^28^. Indeed, rodent studies show that, exposure to stress results in a series of morphological changes, including neuronal atrophy and loss^29,30^. Taken together, these human and rodent studies indicate that depression has some similarities to neurodegenerative diseases that involve neuronal and structural atrophy^31^. It is therefore logical to speculate that approaches that counter neuronal atrophy in the PFC and hippocampus might be potentially effective antidepressant treatments^19,29^. Future studies could target restoration of neural impairments and neuronal atrophy. The exact relationship between specific morphological changes and symptoms remains to be elucidated, however. These structural brain alterations might be neuroanatomical markers of current depressive state, and reversible, or predisposing factors that predate the onset of depression and be relatively difficult to reverse^22,26^. Determining the exact role of changes in synaptic plasticity in depression, and responses to antidepressant treatments, is an important goal of ongoing research.

### Synaptic plasticity and rapid-acting antidepressant

With this in mind, the discovery of a rapid-acting antidepressants offers new insights into the pathogenesis of depression and the role of altered neuroplasticity in antidepressant effects. Ketamine is an N-methyl-d-aspartate (NMDA) receptor antagonist and widely used as a surgical anesthetic^32^. Ketamine exerts a rapid antidepressant effect in refractory depression patients (those responding poorly to traditional antidepressants). Berman and colleagues found that a low-dose of ketamine could exert antidepressant effects within hours that startlingly persisted for several months^33^. Basic science studies in animal models showed that the rapid antidepressant actions of ketamine might correlate with stimulation of synaptic structural plasticity and synaptogenesis^29,34^.

Synaptic plasticity, the most important and basic functional form of neuroplasticity, is acknowledged to play a fundamental role in the neurological pathogenesis of several neuropsychiatric diseases^35,36^. Basic science studies have focused on the underlying cellular and molecular mechanisms of ketamine’s rapid-acting antidepressant effects. Ketamine blocks NMDA receptors located on inhibitory gamma-aminobutyric acid (GABA)-ergic interneurons, resulting in a disinhibition of pyramidal cells of hippocampus^37^. Consequent release of glutamate activates the α-amino-3-hydroxy-5-methyl-4-isoxazolepropionic acid (AMPA) receptors, resulting in depolarization, influx of calcium, and stimulation of post-synaptic neurons^38^. Notably, the depolarization and calcium influx stimulate the release of the brain-derived neurotrophic factor (BDNF), which subsequently activates the Tropomyosin receptor kinase B (TrkB) receptor, and the mammalian target of rapamycin complex 1 (mTORC1) signaling pathway. mTORC1 can potentiate the synthesis of synaptic proteins are important for synaptogenesis and spinogenesis^39,40^. Since the treatment with the selective mTORC1 inhibitor rapamycin can block ketamine’s rapid antidepressant actions, this establishes a critical regulatory role of mTORC1 in these effects^41^. Altered synaptic plasticity produced by antagonism of the glutamate receptor NMDA subtype on GABAergic interneurons is a likely mechanism for ketamine’s rapid-acting antidepressant effects, but more work is needed to further confirm the adaptive significance of synaptic plasticity in all of these contexts: in response to chronic stress or resulting from other factors contributing to depression, as well as traditional and fast-acting antidepressants.

### The impact of BDNF on neuroplasticity in depression

The classic neurotrophic hypothesis of depression presents that depression risk factors and antidepressant treatments can affect the development of depression, respectively, via decreasing or increasing the expression of neurotrophic factors, in particular BDNF^3^. BDNF plays a significant modulatory role in neuroplasticity, neurogenesis, and neuroprotection, and is the most widely reported neurotrophic factor implicated in pathogenesis of depression^42,43^. In acute and chronic stress models, rodents exhibit decreased expression of both BDNF mRNA and protein in the PFC and hippocampus^44^. Antidepressant treatments, including SSRIs, and non-specific monoamine reuptake inhibitors, reverse the reduction of hippocampal BDNF which accompanies chronic stress, resulting in reduced depressive behaviors^43^. Clinical research has shown that blood BDNF levels negatively correlate with the severity of depressive symptoms^45,46^.

Recent studies investigating changes in neural plasticity associated with stress and depression suggested that BDNF might contribute to functional and structural adaptions of synapses in depression-related brain regions. As mentioned above, BDNF activates TrkB receptors and induce activation of downstream phosphoinositide 3-kinase (PI3K)/Akt and mitogen-activated protein kinases (MAPK)/ERK signaling pathways, both of which target the mTORC1 signaling pathway^47^. These pathways regulate several neuronal functions, including synaptic protein synthesis contributing to the formation and development of synapses^48^. Long-lasting stress can lead to synaptic loss, reduction of dendrite spine density, neuronal atrophy and apoptosis, all of which are related to inhibition of BDNF signaling pathways^48^. Consistent with that finding, it was found that heterozygous BDNF knockout rats, with reduced BDNF expression, display greater stress-induced decreases in spine density and dendrite length in the hippocampus and PFC.^49^^.^ These findings suggest that BDNF expression protects against stress-induced deficits in neuroplasticity that are involved in depression and that differences in BDNF expression might underlie some part of individual differences in resilience and vulnerability to stress-induced psychiatric disorders. Thus, there might be an etiological link between altered BDNF and impaired neuroplasticity in depression. Moreover, typical antidepressants fail to stimulate glutamate release rapidly and subsequent activation of BDNF-TrkB signaling, which may contribute to the low efficacy of these drugs^31^. Thus, differences in this response, between individuals, as well as between treatments (e.g. traditional versus fast-acting antidepressants), might contributed to therapeutic efficacy.

## Leptin and depression

### Human studies

As summarized in Table 1, a number of clinical studies have investigated the link between leptin and depression. However, these studies investigating the relationship of MDD and leptin levels have yielded somewhat discrepant results. Initial studies reported lower leptin levels in depressive patients compared to healthy controls, which coincided with initial reports of leptin’s antidepressants effects in animal models of depression^50–53^. However, higher leptin levels in MDD have also been reported (see Table 1).

**Table 1:** Overview of measurements of leptin function in depression

Subjects	Sex (F/M), *N*%	BMI	Assessment	Source and assay	Main findings	Refs.
Control	301/196, 60.6	25.1 ± 4.5	DSM-IV	Fasting blood; ELISA	Higher leptin was associated with the atypical MDD subtype both for remitted and current patients	^60^
Remitted MDD	480/231, 71.0	25.8 ± 4.9				
Current MDD	754/308, 67.5	25.8 ± 5.3				
Control	16/26	25.61 ± 3.50	HDRS	Fasting serum, ElISA	Higher leptin was found in patients with ADD than in controls, but not in patients with DD-NA	^59^
ADD	16/26	26.36 ± 4.17				
DD-NA	20/31	24.41 ± 3.43				
Minimal to no depression	60	29 ± 7	BDI-II scores 0–13	Fasting serum, ElISA	Leptin levels increased with the increasing BDI-II score; participants with moderate to severe depression had the highest levels of leptin	^62^
Mild depression	67	31 ± 9	14–19			
Moderate to severe depression	63	33 ± 8	20–28			
Control	44	24.2 ± 3.7	DSM-IV	Fasting plasma, ELISA	Women with melancholic depression had higher leptin levels than controls; no changes were observed between undifferentiated or atypical patients and controls	^61^
Undifferentiated	22	27.2 ± 6.2				
Atypical	16	28.9 ± 7.1				
Melancholic	51	25.8 ± 6.0				
Control	26/25	16.9 ± 2.0	DSM-IV,BDI,BPRS	Fasting plasma, ELISA	Lower leptin and cholesterol levels in patients with MMD than controls.	^53^
Schizophrenia	39/39	24.1 ± 2.8				
MMD	35/34	20.6 ± 2.7				
Healthy	9	23.43 ± 3.33	DSM-IV	Fasting serum, ElISA	Women had higher leptin levels than men in both depressive and healthy subjects; female patients had significantly higher leptin levels compared to the control females both before and after the treatment	^57^
Healthy female	14	26.13 ± 2.56				
Male MMD patients	12	23.92 ± 3.58				
Female MMD patients	20	26.74 ± 4.88				
Control	69/34, 67	26.0 ± 5.5	DSM-IV	Fasting serum, ElISA	Patients with BD present lower leptin levels than those with MD and the control group.	^101^
Current depression	69/34, 67	26.5 ± 4.9				

*MDD* major depressive disorder, *ADD* depressive disorder with atypical features, *DD-NA* depressive disorder without atypical features, *UD* unipolar depression, *BD* bipolar depression

Depressive patient subjects in clinical studies differ in selection criteria and recruitment strategies, which result in differences in study populations that could confound studies, such as those discussed above. Potential confounding factors include differences in age, gender ratios, fat content and other metabolic factors, medication history, and subtypes of depression. The gender ratio of depressive and healthy subjects is a key factor that may address studies examining the role of leptin in depression. Female serum leptin concentrations are higher than men under normal physiological conditions^54^, and females also have higher rates of depression than males^55,56^. Esel and colleagues reported that female patients with MDD exhibited higher serum leptin levels than non-depressed females, while males had lower levels with or without a diagnosis of depression, which had no effect on leptin levels in males^57^ Therefore, the proportion of female participants in depressive and healthy subjects within a study could affect the observation of differences when investigating the association between leptin and depression.

Depression is a mood disorder with complicated etiopathogenesis, with different subtypes (or variable endophenotypes) which differ across individual patients. This complexity is in addition to such major divisions among mood disorders as MDD, bipolar disorder and seasonal affective disorder^58^, Gecici and colleagues first investigated the relationship between leptin and different clinical subtypes of MMD and found that depressive patients with atypical symptoms had higher serum leptin levels than controls^59^. No differences were found between depressive patients with a more typical profile and controls^59^. That leptin levels are higher in patients with atypical MDD, distinguished by significant weight gain and hyperphagia, was confirmed in a recent study^60^. Contradictory results exist, including a study showing that female patients with melancholic features have higher leptin levels than controls, but this study had a small sample size that may be influenced by the confounding factors previously mentioned^61^. In any case, positive correlations between the severity of depression and leptin levels were observed. Serum leptin levels in patients with moderate to severe depression were higher than those with mild or minimal depression^62^. Noting that those depressive patients with higher leptin levels also had elevated BMI, it is hard to conclude that leptin is positively linked to depression, rather than a compensatory effect induced by adiposity and metabolic syndrome.

A recent meta-analysis of studies comparing adipokines in MDD found no significant associations between peripheral leptin and MDD diagnosis^63^. Thus, based on the current results, leptin is not a biomarker for the diagnosis of depression or other mood disorders. In fact, the hyperleptinemia that has been observed in some cases of depression seems to associated more with central leptin resistance induced by adiposity and metabolic abnormities characterized by impaired leptin sensitivity. Given that mood disorders and metabolic syndromes co-occur and might interact with each other during their pathophysiological processes, increased leptin could be seen as an indirect proxy of metabolic abnormities attached to depression, rather than a pathological mediator. This could explain why elevated circulating leptin was detected most strongly in patients with atypical MDD.

### Animal studies

As summarized in Table 2, several studies have examined stress-treated rodents or genetically modified rodents as depression models to investigate the link between leptin and depression. Initial studies found that exposure to chronic unpredictable stress (CUS) or chronic social defeat stress (CSDS) decreased leptin levels both in plasma and serum^64,65^. Leptin supplements, including peripheric leptin administration^64,66^ or transgenic overexpression of leptin^67^, could reverse behavioral deficits induced by chronic stress. These results indicate potential antidepressant actions of leptin, and implicate leptin deficiencies in the pathophysiological mechanisms of depression. Studies in mice with genetic deletions of the leptin gene and the LepRb gene further confirmed the critical role of leptin in depression. LepRb (db/db) mice^68,69^ and Lep (ob/ob) mice^67^ display depression-like behavioral deficits (see Table 2). Leptin was reported to stimulate the glycogen synthase kinase (GSK)-3β/β-catenin signaling pathway and reverse the inhibitory effects of a glucocorticoid receptor agonist on β-catenin, indicating that GSK-3β/β-catenin signaling pathway-dependent neurogenesis is implicated in leptin’s antidepressant effect^66^. In addition, it is notable that leptin can affect the BDNF concentrations and subsequently the BDNF/TrkB signaling pathway. Based on much of the foregoing discussion, this would be a likely mechanism by which leptin might exert the antidepressant actions^67^.

**Table 2:** Overview of Leptin’s effect on depression in experimental models

Model	Behavioral assessment	Main findings	Mechanism	Refs.
CUS rats CSDS rats	FST	CUS rats and CSDS rats displayed low leptin levels in plasma. Systemic leptin administration reversed the CUS-induced hedonic-like deficit and improved behavioral despair dose-dependently		^64^
CSDS mice	EPM, FST	Stressed mice have lower serum leptin levels than controls. CSDS mice displayed central leptin resistance; administration of a melanocortin agonist worsens stress-induced behavioral deficits, while mice lacking the melanocortin 4-receptor display attenuated symptoms	β3-adrenergic receptors	^65^
Lep (ob/ob) mice LepTg mice DIO mice	FST, SPT	Excess leptin reversed depression-like behaviors; leptin failed to induce an antidepressant action or alter c-Fos expression in the hippocampus of DIO mice, but significantly increased hippocampal BDNF concentrations in CD mice	TrkB/BDNF signaling pathway in hippocampus	^67^
LeprDAT-Cre mice (LepRb was selectively deleted in dopamine neurons.)	SPT, FST, TST	LeprDAT-Cre mice displayed an anxiogenic-like phenotype, while depression-like behaviors were not affected; microinjection of the D1 antagonist SCH23390 into the CeA attenuated the anxiogenic phenotype	DA signalings in midbrain	^70^
CUS rats	OFT, SPT, FST	Leptin administration reversed the CUS-induced reduction of hippocampal neurogenesis and depression-like behaviors; leptin increased β-catenin and reversed the inhibitory effects of dexamethasone on β-catenin	GSK-3β/β-catenin signaling-dependent neurogenesis	^66^
LepRb cKO mice (LepRb was ablated in glutamatergic neurons of forebrain)	FST, TST, RT, SPT, LHT, Hot-plate test, EPM, OFT, LDT	Lepr cKO mice displayed depression-like behavioral deficits loss of Lepr in forebrain glutamatergic neurons facilitated NMDA-induced hippocampal LTD	NMDA-mediated LTD	^73^
LepRb (db/db) mice	SPT, FST, TST, LA	LepRb (db/db) displayed resistance to treatment with either fluoxetine or desipramine; fluoxetine failed to stimulate phosphorylation of Akt(Thr308) and GSK-3β(Ser9) in the hippocampus and PFC of db/db mice	Akt/GSK signaling	^68^
LepRb (db/db) mice	SPT, FST, TST, OFT	LepRb (db/db) mice displayed depression-like behaviors; STAT3 activity and phosphorylation at Tyr 705 were decreased by LepRb KO, which did not involve iIKKb/NFjB signaling	STAT3/SOCS3 signaling	^69^

*CSDS* chronic social defeat stress, *CUS* chronic unpredictable stress, *LA* locomotor activity, *TST* tail suspension test, *FST* forced swim test, *SPT* saccharin preference test, *LHT* learned helplessness test, *EPM* elevated plus-maze, OFT open-field test, *LDT* light dark test, *RT* rotarod test, LepTg mice (transgenic skinny mice overexpressing leptin in the liver), *LTD* long-term depression, DIO diet-induced obesity

## Role of leptin in neuroplasticity and depression

### Leptin and hippocampal synaptic plasticity

Leptin was reported to modulate hippocampal transmission synaptic efficacy, including long-term potentiation (LTP) and long-term depression (LTD). Different doses of leptin, and other circulating hormones affected by leptin, affect LTP and LTD, but pharmacological studies investigating how leptin affects hippocampal synaptic function have shown inconsistent results. Administration of an intermediate dose of leptin directly into the hippocampus stimulates LTP and the hippocampal-dependent learning and memory; however, both higher and lower doses of leptin inhibit LTP^71^. Moult et al. found that leptin can reverse LTP at hippocampal CA1 synapses, in a time and concentration-dependent manner^72^. A competitive NMDA receptor antagonist can block this effect. Leptin-induced LTP preferentially occurs in the hippocampus of adult animal models. This may involve activation of GluN2A containing NMDA receptors and AMPA receptor trafficking^74^. A recent study examined conditional knockout mice in which LepRb was ablated selectively in glutamatergic neurons in the hippocampus and PFC (LepRb cKO mice). This selective ablation of LepRb produced depression-like behavioral deficits and facilitation of NMDA receptor-induced LTD in the hippocampus. Interestingly, LepRb cKO mice are highly sensitive to Ro25-6981, an NMDA-type glutamate receptor subunit (GluN2B) antagonist, which produced antidepressant effects, but resistant to leptin. This finding suggests that leptin modulates excitatory synaptic strength via stimulating NMDA receptors^73^.

**Fig. 1:**
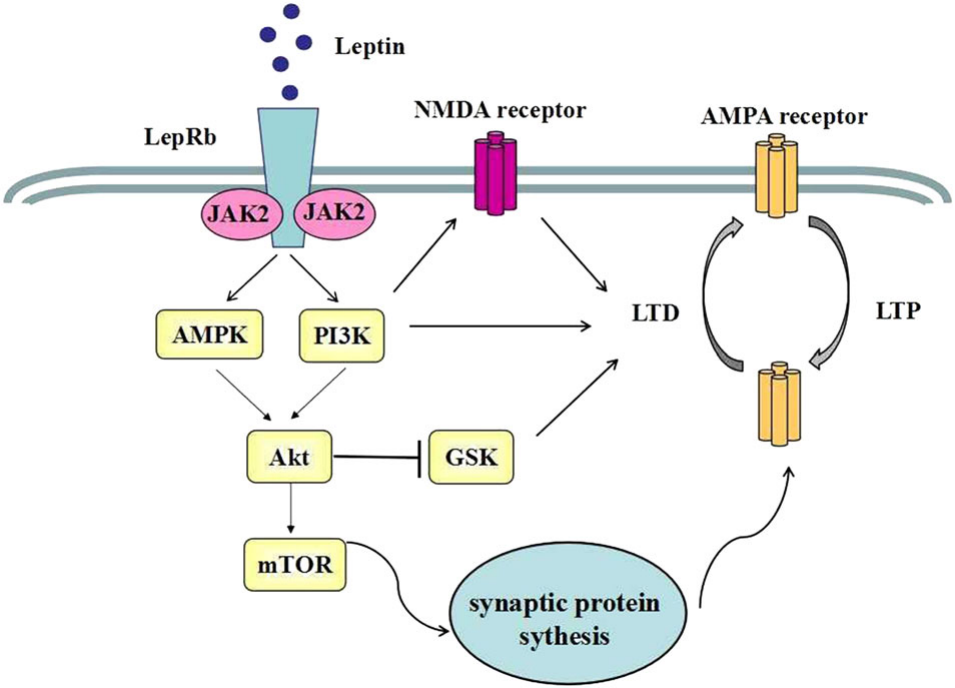
Leptin’s effect on hippocampal synaptic plasticity. Activation of LepRb can trigger PI3K/Akt and AMPK/Akt signaling. Both pathways can subsequently inhibit GSK3β activity. PI3K stimulation can enhance NMDA-dependent LTD and stimulate AMPA receptor exocytosis, which contributes to potentiation of excitatory synaptic transmission. Another critical event is mTOR activation, which promotes a synthesis of synaptic protein synthesis and synaptogenesis

Leptin-induced facilitation of LTP is consistent with effects on learning and memory, but these may extend to other explain antidepressant effects of leptin. Glutamate dysfunction plays a major pathological role in depression, and other stress-related mood disorders^75^. Acute or chronic stress exposure increases glutamate release in limbic brain regions^76^. Adaptations to these stress-induced increases may connect stress to subsequent dysfunctional states, or amelioration of these states by antidepressant treatments. For example, tail suspension evokes a rapid increase in glutamate release in the hippocampal CA3 region and peripheral leptin injections decrease stress-evoked glutamate release^77^. Leptin' effect on glutamate may involve mTOR1 signaling, implicated in the effects of fast-acting antidepressants. mTOR plays a critical role in synaptic protein synthesis, and has been implicated in stress, depression, and antidepressant responses. Rapid-acting antidepressants stimulate in mTOR1 expression and facilitate mTORC1-dependent synaptic plasticity. Emerging evidence indicates that leptin can activate the PI3K/mTOR pathway in neuronal cells^78^. Activation of LepRb can stimulate mTOR activation and consequently promotes synthesis of synaptic proteins contributing to the maturation of old synapses and the formation of new synapses^16^ (see Fig. 1).

### Leptin and neurogenesis

**Fig. 2:**
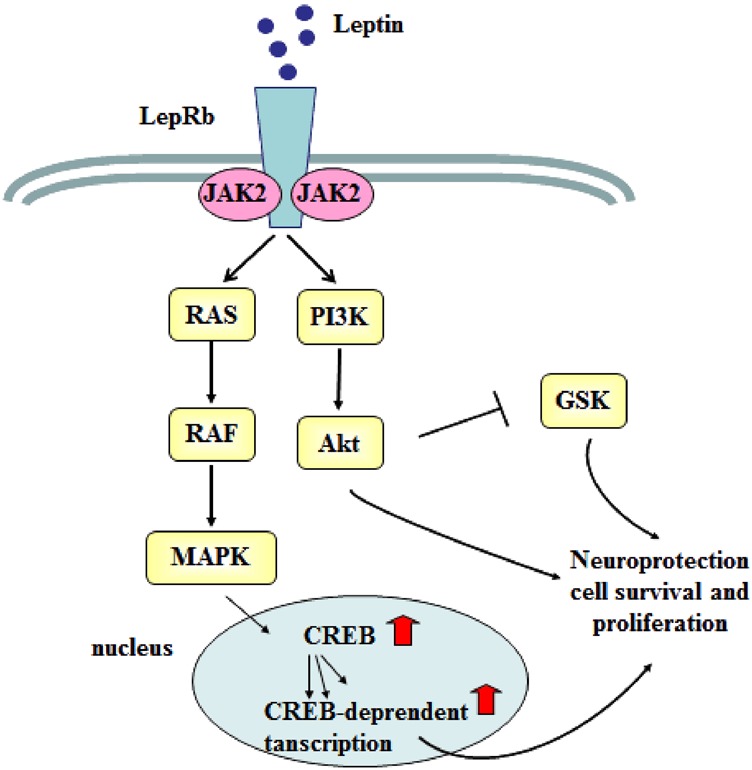
Leptin activates multiple signaling pathways that potentiate neuroprotection, cell survival, and proliferation. Leptin binds to LepRb and activates the PI3K/Akt signaling pathway. Activating LepRb also stimulates the MAPK-signaling pathway. Both pathways promote neuroprotection, cell survival, and proliferation. The stimulation of PI3K/Akt decreases GSK-3β activity by phosphorylating GSK-3, promoting the expression of cell proliferation genes. Activation of the LepRb leads to CREB phosphorylation and initiation of CREB-dependent transcription, which also enhances cell proliferation genes. CREB cAMP response element-binding protein

In addition to the data implicating leptin in synaptic neuroplasticity, accumulating evidence also shows that leptin plays a critical role in neurogenesis. This includes stimulating synaptogenesis and neurogenesis in the hypothalamus^79^, but also extends to other brain regions where leptin induction of neurogenesis might underlie the antidepressant effect of leptin. Leptin can reverse the chronic stress-induced and glucocorticoid-induced inhibition of hippocampal neurogenesis and normalize behavioral deficits by activating the GSK-3β/β-catenin signaling pathway^66^ (Fig. 2). Ser9 phosphorylation inhibits the intracellular regulator GSK-3β, while Tyr216 phosphorylation stimulates GSK-3β^80^. LepRb (db/db) mice have reduced Ser9 phosphorylation and a disinhibition of GSK-3β activity. Leptin inhibits GSK-3β activity via Ser9 phosphorylation in rats^66^. Leptin could inhibit GSK-3β activity and subsequently increase β-catenin signaling via activating the Akt signaling pathways^69^. Increased GSK-3β/β-catenin signaling might induce neuroprotection, cell survival, and proliferation^81,82,83^, which all relate to the proposed pathophysiological mechanisms of depression. In support of this conclusion, GSK-3β and β-catenin activity in the brain is associated with susceptibility to stress-related mood disorders^84,85^.

Previous studies reported that LepRb(db/db) mice, which lack functional LepRb, have reduced spine density on dentate granule cells of the hippocampus^86,87^. Dhar et al. further suggested that leptin can stimulate functional dendrite spines and the formation of synapses in CA1 and CA3 neurons via the MAPK/ERK pathway and the subsequent induction of cAMP-response element binding protein (CREB)-dependent transcription^88^. CREB is an essential regulatory protein for cell survival, neuroprotection, and other neurotrophic factor-dependent synaptic events. That this signaling pathway is also regulated by leptin might indicate another mechanism by which leptin could regulate neurogenesis. Leptin also plays a significant role in the development of the cerebral cortex. LepRb mRNA exhibits high expression during cortical development and leptin deficiency could lead to a reduced number of cortical neurons during the early embryonic life^89^. It remains to be seen whether this also might implicate a role for leptin in cell survival in the adult cerebral cortex.

### Potential neurotrophic effects of leptin and BDNF

Emerging evidence from human and animal studies demonstrates that leptin plays a potential neurotrophic role in the central nervous system (CNS). Leptin exerts neuroprotective actions and inhibits the dysfunction induced by a variety of neurotoxic insults, including accumulation of amyloid-β, exposure to excitotoxic glutamate, and ischemia^90,91,92^. Alhough the specific molecular mechanisms of leptin’s neuroprotective effects are still obscure, the activation of downstream Jak2/STAT3, MAPK/ERK, PI3K/Akt, STAT5, and SHP-2/SOCS3 pathways might be involved. Leptin replacement therapy in leptin-deficient patients significantly increases the gray matter concentration in the anterior cingulate gyrus, cerebellum, and inferior parietal lobule^93^. This indicates that leptin either has somewhat broad effects on aspects of neural plasticity, neural proliferation, or neuroprotection. These effects have functional consequences; leptin supplement-induced structural changes detected in hippocampus and cortex improve cognition^94^.

Growing evidence demonstrates a link between BDNF expression and regulation of metabolic balance. Food deprivation, characterized by lower leptin secretion, can reduces BDNF expression within the dorsal vagal complex (DVC) and hippocampus^95,96^. Leptin stimulates BDNF mRNA translation in the dendrites of hypothalamic neurons^97^. Hippocampal BDNF expression is decreased in LepRb-deficient mice^98^. We have highlighted above that leptin can stimulate BDNF secretion via GSK-3β/β-catenin and MAPK/ERK pathways. These results implicate leptin signaling in the synthesis and expression of BDNF, and consequently in BDNF-dependent functions, which includes depression and responses to antidepressant drugs.

Mice with diet-induced obesity have decreased hippocampal BDNF expression and behavior indicative of a depressive state^67^. Leptin treatment fails to increase hippocampal BDNF concentrations due to the impairment of leptin responses, which is known as leptin resistance induced by obesity^67^. The antidepressant effects of leptin thus appear to be mediated by activation of BDNF systems in hippocampus. These data further suggest that leptin responsiveness may be a biomarker, in some cases, of a depressive state associated with this mechanism. Moreover, reversal of leptin resistance by itself might restore normal BDNF function, and consequent effects on depressive states. Overall, these data implicate leptin and BDNF in the regulation of energy homeostasis and depression, and suggest that there is substantial crosstalk between these physiological functions, which should not be surprising given some of the core symptoms of depression (that may include lethargy and changes in appetite). Leptin is therefore a neurotrophic factor, albeit perhaps an indirect one, although the specific cellular and molecular mechanisms underlying the effects of leptin discussed here, that are BDNF-dependent and BDNF-independent, require further investigation.

## Conclusion

Neural plasticity is the capacity of the brain to undergo functional and/or structural adaptation in response to a range of intrinsic and extrinsic stimuli. The relationship between alterations in neural plasticity and depression, as well as the response to antidepressants, is not yet fully understood, but has been widely implicated by preclinical and clinical studies. Moreover, studies of fast-acting antidepressants are helping to illuminate the place of neural plasticity in depression and antidepressant effects. Ketamine rapidly increases the number of synaptic connections and reverses synapse loss resulting from chronic stress and depression. The emergence of ketamine as a novel antidepressant agent further supports the idea that synaptic plasticity is involved in the pathophysiology of depression and antidepressant responses. Leptin plays a vital role in neuroplasticity, especially in depression-related regions of the brain. Leptin can influence neuronal morphology and hippocampus synaptic transmission, and may act as a neurotrophic factor, if not directly, then indirectly via BDNF or other factors. Moreover, other neuropeptides, such as galanin, ghrelin, and corticotropin-releasing factor, have also been implicated in depression^99,100^. Consequently, leptin are potential therapeutic targets for treatment of depression, and elucidation of leptin-associated mechanisms underlying neural plasticity will enhance our understanding of the psychopathology of depression.
